# Evaluation of the speed of kill of a novel orally administered combination product containing sarolaner, moxidectin and pyrantel (Simparica Trio™) against induced infestations of *Ixodes scapularis* on dogs

**DOI:** 10.1186/s13071-020-3953-2

**Published:** 2020-03-01

**Authors:** Susan Holzmer, Kristina Kryda, Sean P. Mahabir, William Everett

**Affiliations:** 1 Zoetis, Veterinary Medicine Research and Development, 333 Portage Street, Kalamazoo, MI 49007 USA; 2 BerTek, Inc., P.O. Box 606, Greenbrier, AR 72058 USA

**Keywords:** Dog, Moxidectin, Oral, Pyrantel, Isoxazoline, *Ixodes scapularis*, Sarolaner, Simparica Trio™, Speed of kill, Tick

## Abstract

**Background:**

The black-legged (or deer) tick, *Ixodes scapularis*, commonly infests dogs in the USA and is the vector of important zoonotic pathogens, including *Borrelia burgdorferi*, the causative agent of Lyme disease. Rapid onset of activity is important in reducing the feeding activity of ticks, thereby reducing the possibility of transmission of infections. The speed of kill of a novel oral combination product, Simparica Trio™ containing sarolaner, moxidectin and pyrantel was evaluated in a well-controlled laboratory study against an existing infestation and subsequent weekly induced infestations of *I. scapularis* ticks on dogs.

**Methods:**

Dogs were allocated randomly based on host suitability tick counts to treatment with a single dose of either placebo or Simparica Trio™ at the minimum label dose of 1.2 mg/kg sarolaner, 24 µg/kg moxidectin and 5 mg/kg pyrantel (as pamoate salt). All dogs were infested with approximately 50 unfed adult *I. scapularis* ticks at a 1:1 sex ratio on Days −2, 7, 14, 21, 28 and 35. Tick counts were conducted at 8, 12 and 24 h after treatment on Day 0 and after each subsequent infestation.

**Results:**

No treatment-related adverse events occurred during the study. Dogs in the placebo-treated group maintained adequate tick infestations for the duration of the study. Day 0 tick counts at 8 h after treatment with Simparica Trio™ were reduced relative to placebo against an existing infestation with efficacy of 67.5%, demonstrating that Simparica Trio™ started killing ticks soon after treatment. Efficacy was 98.4 % at 12 h and 99.4% at 24 h. Rapid speed of kill was maintained throughout the month, with efficacy of ≥ 94.2% at 24 h after re-infestation through Day 28.

**Conclusions:**

A single dose of Simparica Trio™ administered orally to dogs at the minimum label dose of 1.2 mg/kg sarolaner, 24 µg/kg moxidectin and 5 mg/kg pyrantel (as pamoate salt) was safe and began to kill existing *I. scapularis* ticks within 8 h after treatment and resulted in ≥ 94.2% efficacy within 24 h against re-infestations for a month.

## Background

Tick infestation is problematic in dogs due to local irritation, alopecia and even anemia in extreme infestations [1]. Ticks are critically important in veterinary medicine due to their ability to transmit a variety of pathogens, some of which cause debilitating and even life-threatening diseases [1, 2].

Following attachment, which can take up to 24 h, the tick begins feeding first in the slow feeding phase, lasting 3 to 5 days [3]. Approximately 12 to 36 h prior to the finish of the blood meal, ticks undergo a rapid growth of the cuticle, when the rapid feeding phase begins and most of the blood meal is consumed by the tick [4]. A single female *Ixodes scapularis* may consume as much as 0.5 ml of blood during the feeding process, and a heavy tick infestation can result in significant blood loss [3]. A product that kills ticks before they have a chance to consume a significant blood meal is critical to canine health. Tick-transmitted infections require a period of activation before the tick is capable of transmitting them *via* the salivary glands. This activation period begins when the tick starts to bite the host, and ixodid ticks require at least 24 h to attach for the successful transmission of most tick-borne pathogens [5–9].

Rapid parasite killing is important in reducing the feeding activity of ticks, thereby reducing the probability of transmission of pathogens such as *Anaplasma*, *Babesia* and *Borrelia burgdorferi*, which are important in dogs and are also zoonotic infections transmitted by *I. scapularis*. If infected ticks are killed within 24 h, transmission of these pathogens may be prevented [10–12].

*Ixodes scapularis* commonly infests dogs in the USA and is the vector of important zoonotic pathogens, including *Borrelia burgdorferi*, the causative agent of Lyme disease. *Borrelia burgdorferi* is transmitted when the tick injects this bacterial spirochete into the host during the bite event, beginning between 24 and 36 h after the tick attaches to its host [5]. *Ixodes scapularis* is also the vector for *Anaplasma phagocytophilum*, which causes anaplasmosis in humans and dogs, *Ehrlichia* spp., causing ehrlichiosis in dogs and humans, and *Babesia microti*, which cause babesiosis in humans [12–17].

Sarolaner belongs to a potent new class of ectoparasiticides (isoxazolines) and has been shown to provide consistent efficacy against fleas and ticks in Simparica^®^ and Simparica Trio™ for 1 month following a minimum single oral dose of sarolaner at 2.0 and 1.2 mg/kg respectively [18–20]. Sarolaner exerts activity against invertebrates by blocking GABA-gated chloride channels [21, 22]. In addition to sarolaner, moxidectin has been included in Simparica Trio™ at a minimum dose of 24 µg/kg to provide robust protection against lungworm and heartworm [23, 24]. Pyrantel is also included to provide treatment and control of roundworms and hookworms [25, 26]. Combining these three ingredients in an all-in-one chewable product provides a convenient option for owners whose dogs require protection from ectoparasites, heartworm and/or lungworm disease and intestinal parasites.

This laboratory study was conducted to assess the speed of kill of *I. scapularis* by the combination product (Simparica Trio™, Zoetis, Parsippany, NJ, USA) at the minimum label dose of 1.2 mg/kg sarolaner, 24 µg/kg moxidectin and 5 mg/kg pyrantel (as pamoate salt).

## Methods

The study was conducted in accordance with the World Association for the Advancement of Veterinary Parasitology (WAAVP) guidelines for evaluating the efficacy of parasiticides for the treatment, prevention and control of flea and tick infestation on dogs and cats [27] and complied with Good Clinical Practice guidelines [28]. Masking was accomplished by separation of functions of study personnel. All personnel who made study observations, conducted tick infestations and tick counts and/or categorizations or performed general care for the dogs were masked to experimental treatments.

### Animals

All dogs used in the study demonstrated good tick retention prior to treatment and were in good health at treatment. Fifty-four Beagles and mixed-breed dogs, 28 males and 26 females aged 7–42 months and weighing 5.5–13.6 kg, were included in the study. Each animal was individually and uniquely identified by ear tattoo or microchip. Dogs were housed in indoor, individual runs that ensured no physical contact between dogs. The animals were fed a commercial dog food to provide a maintenance diet and fresh water was available *ad libitum*. Cross-contamination between dogs was prevented by using separate equipment and by changing the protective clothing of the personnel handling each dog.

### Design

The day of treatment was designated as Day 0. The animals were acclimated to the study housing beginning 14 days prior to treatment and general health observations were performed at least once a day from the start of acclimation period until the end of the study. On Day −7, dogs were examined to ensure they were free of ticks and were each then infested with approximately 50 *I. scapularis* ticks (1:1 sex ratio) to determine host suitability and allow the selection of study dogs from the pool of 64 available animals. On Day −5, the dogs were examined to count and remove the ticks. The 54 dogs retaining the highest numbers of live ticks were selected for inclusion in the study. The dogs were ranked by decreasing tick counts in blocks of six and randomly allocated within block to six groups of nine dogs each. One group of placebo-treated dogs and one group of combination product-treated dogs were assigned each to tick count at 8, 12 or 24 h after treatment and subsequent tick infestations. Blocks of animals were randomly assigned to adjacent pens within the test facility. Dogs were weighed and moved into their allocated pens on Day −4.

The dogs were infested with *I. scapularis* ticks on Day −2. Prior to treatment on Day 0, all dogs were given a physical examination to evaluate general health and confirm suitability for inclusion into the study. On Day 0, dogs were administered either placebo tablets or the combination product tablets. At each of the 8, 12 and 24 h time points after treatment, one placebo-treated group and one combination product-treated group was examined to count and remove ticks. The dogs were subsequently re-infested with ticks on Days 7, 14, 21, 28 and 35, and the respective groups were examined for tick counting with removal of the ticks at 8, 12 and 24 h after each re-infestation.

### Treatment

On Day 0, dogs were dosed with either placebo tablets containing inert formulation ingredients (vehicle) or combination product tablets (Simparica Trio™, Zoetis). Tablets were provided in varying strengths, such that a combination of tablets could be administered to ensure dogs were appropriately dosed to the minimum end of the proposed label dose range. Each dog received one to three tablets of Simparica Trio™ to provide as close as possible to the minimum label dosage of 1.2 mg/kg sarolaner (actual doses ranged from 1.2 to 1.6 mg/kg), 24 µg/kg moxidectin (actual doses ranged from 24 to 33 µg/kg) and 5 mg/kg pyrantel (as pamoate salt) (actual doses ranged from 5.0 to 6.8 mg/kg). Body weights obtained on Day −4 were used for dose calculation. An equivalent number of placebo tablets, which were similar in appearance to the combination product to maintain masking, were administered to the control animals. Feed was withheld for at least 12 h prior to treatment and animals were not fed again until at least 4 h post-treatment. All doses were administered by hand-pilling to ensure accurate dosing. Each dog was observed after dosing for evidence that the dose was swallowed, and for up to 2 h post-dosing for any signs of emesis. Dogs were examined for general health and any reactions to treatment at 1, 3, 6 and 24 h after treatment.

### Tick infestation and assessment

*Ixodes scapularis* ticks were obtained from the Oklahoma State University tick laboratory colony. The colony was initiated in 1991 with wild-caught engorged females collected locally in Stillwater, Oklahoma, USA. Wild-caught engorged female ticks are introduced into the colony annually. The last introduction of field collected ticks to the colony was about 8 months prior to the study.

For each infestation, 50 (± 5) adult *I. scapularis* with a sex distribution of approximately 1:1 were applied to each dog. Dogs were gently restrained by hand and the pre-counted aliquot of ticks was applied directly onto the dog’s coat and allowed to disperse into the hair.

Tick counts were performed by personnel trained in the standard procedures in use at the test facility. Personnel conducting tick counts were unaware of treatment assignments and dogs were examined in a non-systematic order. Protective gloves and clothing were changed between dogs. Initially, the dog’s entire body was visually examined, pushing the hair against its natural nap to expose ticks that were then counted and removed. After this manual inspection, an extra-fine tooth comb was used to comb the animal thoroughly to remove any otherwise missed ticks. Each dog was examined for a total of at least 10 min. If ticks were encountered in the final minute, combing was continued in 1-min increments until no further ticks were encountered. The ticks were examined to assess viability by assessing movement of the legs due to tactile stimulus or by blowing on them to expose them to CO_2_ in exhaled breath.

### Statistical analysis

The individual dog was the experimental unit and the primary endpoint was live tick counts. Tick counts were transformed by the ln (count + 1) transformation prior to analysis in order to stabilize the variance and normalize the data. Using the PROC MIXED procedure (SAS 9.4, Cary NC), transformed counts were analyzed using a mixed linear model with treatment group as a fixed effect, block within room and error as random effects at each time point. Testing was two-sided at the significance level α = 0.05. Percent efficacy was calculated from arithmetic means using Abbott’s formula for each counting time point:

$${\% } \;{\text{Reduction}}\;{ = }\; 1 0 0\; \times \;\frac{{{\text{Mean count }}\left( {\text{placebo}} \right) \;{-}\; {\text{Mean count }}\left( {\text{treated}} \right)}}{{{\text{Mean count }}\left( {\text{placebo}} \right)}}$$

Tick counts were not conducted for one placebo-treated dog in the 8-h group on Day 7 and two combination product-treated dogs in the 12-h group on Day 21. For this reason, back-transformed least squares means were used rather than arithmetic means which were to be used at these time points.

## Results and discussion

### Efficacy

A single oral dose of Simparica Trio™ provided rapid onset of efficacy against an existing *I. scapularis* infestation with a 67.5% reduction in mean live tick counts within 8 h after treatment, and 98.4% reduction by 12 h after treatment (Table 1). At least six dogs in each of the three placebo-treated groups maintained adequate tick infestations, with tick counts ranging from 12 to 47 throughout the study.

**Table 1:** Arithmetic mean of live tick counts and percent efficacy relative to placebo for dogs treated once orally with Simparica Trio™ at 1.2 mg/kg sarolaner, 24 µg/kg moxidectin and 5 mg/kg pyrantel (as pamoate salt)

Count time^a^	Treatment group	Day^b^					
		0	7	14	21	28	35
8 hours	Placebo	18.1	20.7^d^	21.6	19.1	17.7	17.7
	Simparica Trio™	5.9	8.0^d^	17.1	13.9	17.1	16.8
	% Efficacy	67.5	61.5^d^	20.6	27.3	3.1	5.0
	Test statistic	*t*_(9.71)_ = 2.19	*t*_(6.99)_ = 5.18	*t*_(40)_ = 0.84	*t*_(12.3)_ = 1.29	*t*_*(*13.7)_ = 0.23	*t*_(7.68)_ = 0.75
	*P-*value^c^	0.0545	0.0013	0.4069	0.2191	0.8186	0.4751
12 hours	Placebo	21.3	23.6	18.9	21.2^d^	24.9	19.6
	Simparica Trio™	0.3	3.6	7.7	8.0^d^	11.9	10.9
	% Efficacy	98.4	84.9	59.4	62.2^d^	52.2	44.3
	Test statistic	*t*_(13.2)_ = 18.10	*t*_(9.39)_ = 6.21	*t*_*(*40)_ = 4.00	*t*_(6.6)_ = 4.32	*t*_(13.8)_ = 1.79	*t*_(13.3)_ = 2.27
	*P-*value^c^	< 0.0001	0.0001	0.0003	0.0040	0.0957	0.0404
24 hours	Placebo	17.4	17.2	24.1	16.8	23.1	14.8
	Simparica Trio™	0.1	0.1	0.0	0.3	1.3	4.8
	% Efficacy	99.4	99.4	100	98.0	94.2	67.7
	Test statistic	*t*_(9.29)_ = 9.34	*t*_(9.04)_ = 11.33	*t*_(40)_ = 12.89	*t*_(9.29)_ = 7.98	*t*_(8.03)_ = 9.62	*t*_(9.12)_ = 4.22
	*P-*value^c^	< 0.0001	< 0.0001	< 0.0001	< 0.0001	< 0.0001	0.0022

^a^Time after treatment or subsequent weekly tick infestations

^b^Day of treatment (Day 0) and of subsequent weekly tick infestations

^c^*P-*value for comparison of arithmetic mean tick counts between Simparica Trio™ and placebo groups at each time point within a row, *P* ≤ 0.05 indicates significant difference

^d^Time point with missing observations, back-transformed least squares means and corresponding percent efficacy estimates were used

For subsequent infestations post-treatment, there was a significant reduction in live tick counts at the 8-h time point (*t*_(6.99)_ = 5.18, *P* = 0.0013) on Day 7 only, at the 12-h time point on Days 7, 14, 21 and 35 (2.27 ≤ *t*_*df*_ ≤ 6.21, 6.6 ≤ *df* ≤ 40, *P* ≤ 0.0404) and at the 24-h time point on all days until study end on Day 35 (4.22 ≤ *t*_*df*_ ≤ 12.89, 8.03 ≤ *df* ≤ 40, *P* ≤ 0.0022). Efficacy was ≥ 94.2% at the 24-h time point up to and including Day 28.

As has been shown previously with Simparica^®^ (2 mg/kg sarolaner) [19], in this study Simparica Trio™ demonstrated a rapid speed of kill against existing and subsequent re-infestations of *I. scapularis* in dogs following a single dose at the minimum label dose of 1.2 mg/kg sarolaner, 24 μg/kg moxidectin and 5 mg/kg pyrantel (as pamoate salt). Acaricidal activity occurred at 8 h against an existing infestation as demonstrated by a 67.5% reduction in tick counts. Activity was also seen against subsequent re-infestations at 8 h on Day 7 and at 12 h after re-infestation on Days 7, 14, 21 and 35. Efficacy at 24 h after treatment and re-infestation was > 94% for 4 weeks.

Antiparasitics are an essential tool for veterinarians in the prevention of disease. Owner compliance can be poor when administering multiple preventatives [29] which can put pets at risk of contracting parasites and the pathogens they transmit. Combining sarolaner with pyrantel and moxidectin to provide reliable and effective protection against a broad spectrum of parasites is a convenient alternative to multiple monthly medications. Rapid kill of ticks is important in reducing adverse effects of tick feeding and is critical in the reduction of transmission of tick-borne pathogens. The rapid and consistent efficacy of a single oral dose of Simparica Trio™ shown in this study demonstrates how this treatment may reduce the risk of dogs becoming infected with the pathogens transmitted by *I. scapularis*.

### Safety

There were no treatment-related adverse events observed during the study.

## Conclusions

A single oral dose of the combination product (Simparica Trio™) administered at the minimum label dose of 1.2 mg/kg sarolaner, 24 µg/kg moxidectin and 5 mg/kg pyrantel (as pamoate salt) started killing *I. scapularis* ticks 8 h after treatment administration and provided 98.4% efficacy within 12 h after treatment. Simparica Trio™ resulted in ≥ 94.2% efficacy within 24 h of infestation for a month. Simparica Trio™ will provide a convenient and effective means for treating and controlling this important tick on dogs.

## Data Availability

The dataset supporting the conclusions of this article is included within the article.

## Abbreviations

WAAVP: World Association for the Advancement of Veterinary Parasitology
GABA: Gamma aminobutyric acid
PROC MIXED: Mixed procedure
